# Taming Performance Variability of Healthcare Data Service Frameworks with Proactive and Coarse-Grained Memory Cleaning

**DOI:** 10.3390/ijerph16173096

**Published:** 2019-08-26

**Authors:** Eunji Lee

**Affiliations:** Faculty of Smart Systems Software, Soongsil University, 369 Sangdoro, Dongjak-gu, Seoul 06978, Korea; ejlee@ssu.ac.kr

**Keywords:** medical systems, health data management, data framework for applied health data, database, memory reclamation

## Abstract

This article explores the performance optimizations of an embedded database memory management system to ensure high responsiveness of real-time healthcare data frameworks. SQLite is a popular embedded database engine extensively used in medical and healthcare data storage systems. However, SQLite is essentially built around lightweight applications in mobile devices, and it significantly deteriorates when a large transaction is issued such as high resolution medical images or massive health dataset, which is unlikely to occur in embedded systems but is quite common in other systems. Such transactions do not fit in the in-memory buffer of SQLite, and SQLite enforces memory reclamation as they are processed. The problem is that the current SQLite buffer management scheme does not effectively manage these cases, and the naïve reclamation scheme used significantly increases the user-perceived latency. Motivated by this limitation, this paper identifies the causes of high latency during processing of a large transaction, and overcomes the limitation via proactive and coarse-grained memory cleaning in SQLite.The proposed memory reclamation scheme was implemented in SQLite 3.29, and measurement studies with a prototype implementation demonstrated that the SQLite operation latency decreases by 13% on an average and up to 17.3% with our memory reclamation scheme as compared to that of the original version.

## 1. Introduction

SQLite is an embedded database engine with a rich set of features. Given its efficient and compact design, it is heavily used in mobile applications [1,2,3]. Android is among the most well-known platforms with a high dependency on SQLite, and the web browsers Chrome and Firefox also make use of SQLite to maintain web objects in local storage [1]. Medical devices and healthcare applications employ SQLite for managing the health records [4,5,6,7,8,9] and emerging self-driving car manufacturers also consider SQLite for their in-vehicle database management systems [10]. The popularity of SQLite extends beyond mobile applications; it manifests in various server-side applications such as large-scale data analytics and high-performance bioinformatics, offering high reliability and easy portability in datacenters [6,11].

**Figure 1:**
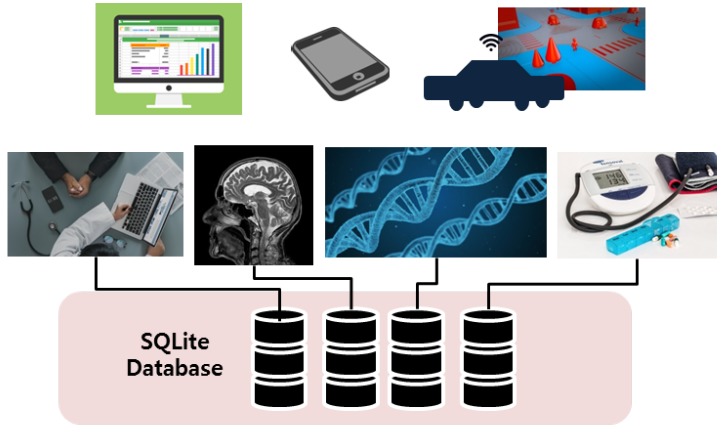
Applications for SQLite.

Despite its growing popularity across various sectors (Figure 1), SQLite falls short of the requirements for a wide range of applications. Natively built around small and lightweight applications, SQLite poses a host of challenges when deployed under many different circumstances. The problem addressed in this study inherently stems from this shortcoming. We observed that SQLite abnormally slows when a large-sized transaction arises, such as storing a magnetic resonance image (MRI) or a high volume of the healthcare data, exporting a long write latency to end users.

Our careful analysis reveals that this performance impairment is primarily associated with the unsophisticated buffer management scheme used by SQLite, which stands in contrast to that employed in a large-scale database management system (DBMS) [12,13,14]. In more detail, SQLite internally maintains a small size of buffer within the main memory; by absorbing large batches of data updates in the buffer and forcing them into storage at once, SQLite offers high throughput for small writes, which are dominant in mobile devices. However, this is not the case for large transactions; SQLite is subject to serious performance slowdowns during large-sized transactions, particularly when they spill out of the buffer. When the buffer runs out of space and is thus unable to accommodate an outstanding transaction, SQLite must reclaim some of the in-use pages in a buffer.

The problem is that SQLite reclaims only one page at a time (i.e., 4 KB). This bit-by-bit behavior of the reclamation scheme causes a significant performance penalty by unnecessarily repeating the costly reclamation routine for every request. Furthermore, this shortcoming exacerbates when the dirty pages should be evicted, which essentially involves storage write to persist the updates after being loaded into memory. In this case, the individual flushing of the dirty pages considerably decreases the I/O efficiency, generating a large number of small writes to storage.

To overcome the aforementioned limitation, this paper presents a new version of SQLite that proactively cleans the dirty pages at a coarse granularity, thereby minimizing undesirable performance delays during large transactions. To this end, SQLite keeps track of the number of dirty pages in the buffer, and when it exceeds a given threshold, SQLite batches them to storage altogether. The threshold is set to a number sufficiently large to render storage writes efficient. For effective realization of this concept, this paper explores two avenues by which to arrive at proactive memory cleaning. The first approach is to rely on a background thread that is likely to seamlessly flush dirty pages in parallel with user-level threads. The second approach is to synchronously perform memory cleaning under the same context of the user threads, which cannot hide the flushing cost from the user-perceived latency but has no inter-thread communication overhead.

We implemented both versions of the proposed scheme in SQLite 3.29, and we evaluated the performance of SQLite using a micro-benchmark. The measurement study with our prototype implementation showed that SQLite with our proactive coarse-grained cleaning scheme outperforms the original version of SQLite by up to 1.177×, providing a performance nearly identical to that with an infinite memory space.

The remainder of this paper is organized as follows. In Section 2, we provide a brief overview of SQLite, while Section 3 explains the design and implementation of the proposed proactive and coarse-grained memory cleaning scheme. Section 4 describes the evaluation methodology and presents experimental results. Section 5 discusses the proposed design in relation to prior work. Section 6 concludes the paper.

## 2. Background

In this section, we present an architectural overview of SQLite, focusing on its transaction handling mechanisms. We then explain four journaling modes that are provided by SQLite.

### 2.1. SQLite Architecture

**Figure 2:**
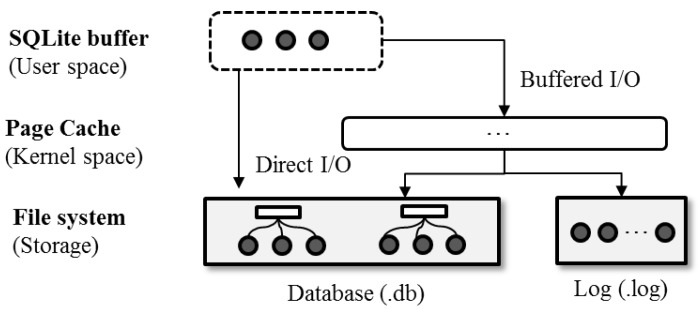
Overall architecture of SQLite.

Figure 2 shows the overall architecture of SQLite. SQLite consists of three key components: an in-memory buffer, a persistent database, and a log. The persistent database and log are maintained as individual files within a file system mounted on non-volatile storage. The in-memory buffer of SQLite resides in the user space of the main memory, running over the page cache managed by the kernel. SQLite inherently uses a B-tree-based data structure to maintain the database in a file [15]. When a data access request arrives, SQLite initially fetches the requested data from the underlying database into the in-memory buffer and then allows users to access the data. SQLite presents different options associated with how the underlying storage device can be assessed. If SQLite opts for the buffered I/O (the default configuration), the data are placed in a page cache before being transferred to the user-level buffer. SQLite can also be set to bypass a page cache and access the database and/or log file directly through the memory mapped I/O.

### 2.2. Journaling Modes in SQLite

SQLite essentially handles all database updates as an individual transaction, ensuring atomicity for each request. Journaling is an integral technique to embody this consistency abstraction in SQLite, and listed below are the journaling modes provided by SQLite [16].

#### 2.2.1. DELETE

In this mode, before altering the database file, SQLite copies the original data into another file called a rollback journal. Once the original data copy is successfully completed, SQLite updates the database file by committing changes into it, after which it deletes the rollback journal at the end of the transaction. If the system crashes during the commit action, SQLite reverts the database to the state before update using the rollback journal.

#### 2.2.2. TRUNCATE

This mode runs similarly to the DELETE mode, but it does not delete the rollback journal file after the commit. Instead, it removes the obsolete data by truncating the file range including them because a file truncate action costs less than a file deletion.

#### 2.2.3. PERSIST

This mode goes further and invalidates the rollback journal file by writing zeros in its header, neither deleting nor truncating it, which is most efficient.

#### 2.2.4. WAL(WRITE-AHEAD LOGGING)

This mode works in an inverted way with regard to the three modes described above. Instead of creating a rollback journal, it maintains a write-ahead log file and writes changes into it, preserving the original data in the database file. All transactions committed in the write-ahead log are eventually reflected in the database file. This operation is called *checkpoint*, and it is invoked when the log file grows in size beyond a configured threshold (e.g., 1000 pages by default).

While the initial version SQLite employs the DELETE mode as a default configuration, SQLite currently operates in the WAL mode by default. Compared to other alternatives, the WAL mode offers a rapid response and high throughput because it quickly logs transactions in the WAL file and efficiently updates the database file with a single large write afterwards.

## 3. Proactive and Coarse-Grained Memory Cleaning

This section explains the design of the proposed proactive and coarse-grained cleaning scheme and presents how to integrate it within the SQLite buffer management system. For the sake of clarity, our explanation assumes that SQLite uses the WAL mode in this section.

### 3.1. Current SQLite Memory Reclamation Scheme

Similar to most databases, SQLite maintains an internal memory buffer that serves to retain recently accessed data in fast store so as to provide high throughput and low latency for subsequent requests [17]. In the case of a write request, instead of forcing a change in storage upon every update, SQLite returns to users only after reflecting the change in the memory buffer, which is an order of magnitude faster. The in-memory buffer is allocated during database creation, with a size of 400 KB by default in SQLite. As opposed to the client/server database system where the memory buffer is shared among processes, SQLite is an embedded database in which each process has a dedicated memory buffer. The buffer space is filled with data over time and eventually is fully occupied. Memory reclamation is invoked when a new transaction arrives at a fully utilized buffer. In this case, SQLite must reclaim an in-use page to accommodate the new data.

**Figure 3:**
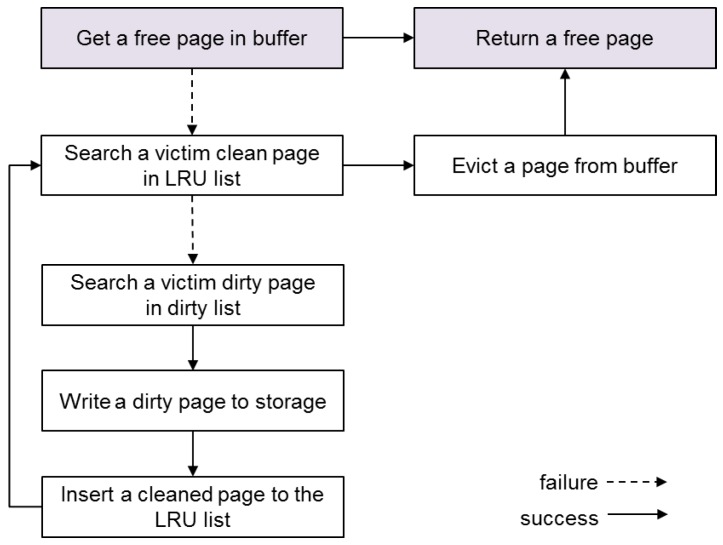
Memory reclamation scheme of SQLite.

Figure 3 describes the current memory reclamation scheme of SQLite. When choosing a victim page for reclamation, SQLite prefers clean pages to dirty pages to avoid expensive storage access during a transaction. Because a dirty page means that the data have been modified after being loaded into memory, the updates should be forced to storage before being evicted from the memory. To this end, SQLite maintains only clean pages in the LRU (Least-Recently-Used) list, while maintaining dirty pages separately in the dirty page list. For reclamation, SQLite first searches the LRU list to find a victim page in a clean state. If it finds any reclaimable page, which has a reference count 0, SQLite frees it by simply initializing its mapping information such that it can accommodate the new transaction data. If it fails to find a victim page in the LRU list, however, SQLite attempts to reclaim dirty pages at the cost of storage write. SQLite searches the dirty page list to find a reclaimable page, and, if any occur, SQLite flushes the data of the victim page into storage before evicting it. Once the data of the victim page are successfully transferred to storage, the dirty page is cleaned and is thus inserted into the LRU list. Then, SQLite goes back to the beginning of the memory reclamation routine and searches the LRU list again for a victim in a clean state.

The drawback of the SQLite buffer management scheme is essentially associated with its piecemeal property in reclaiming the buffer space for recycling. SQLite retrieves the buffer pages one at a time, repeating the aforementioned entire steps of memory reclamation for every request. If multiple pages are eventually requested, this bit-by-bit reclamation imposes serious performance penalties by unnecessarily increasing the software overhead. In addition, when the dirty pages are reclaimed, this shortcoming is exacerbated; the individual flushing of the dirty pages deprives the underlying I/O subsystems for coalescing small writes, significantly decreasing the I/O efficiency. Unfortunately, such a worst-case scenario occurs when a large transaction that spills over the buffer size is given. In this case, the in-memory buffer shortly runs out of space with most of it occupied by the dirty pages. It is inevitable to reclaim dirty pages and a serious performance delay will ensue.

**Figure 4:**
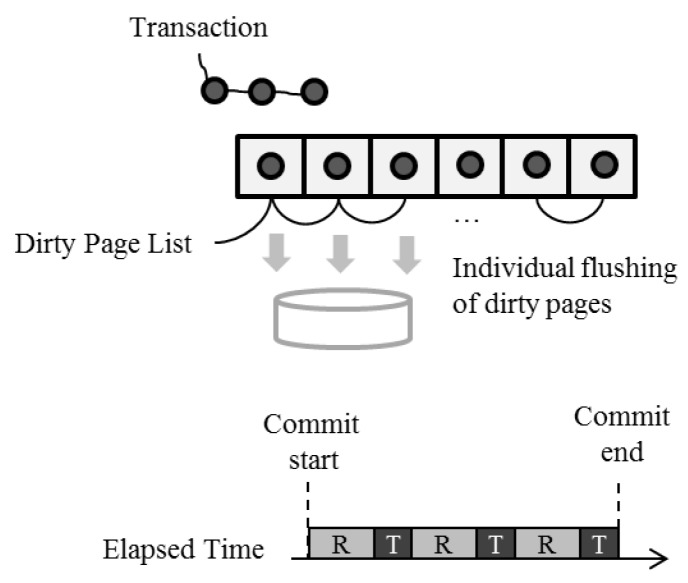
SQLite large transaction processing. The subfigure at the bottom shows the transaction commit timeline. The gray box with R denotes the memory reclamation time duration and the black box with T indicates the data writing time duration of the transaction into the buffer.

Figure 4 shows the large transaction processing of SQLite with a timeline when dirty page reclamation is involved. Writing new data to a buffer alternates with reclaiming a buffer in use. As the small write to storage intermittently continues to occur throughout the entire process, the transaction latency significantly increases.

### 3.2. Proactive and Coarse-Grained Memory Cleaning

Motivated by the above observation, we enhance the current buffer reclamation scheme of SQLite such that it can proactively perform the cleaning of dirty pages in an efficient manner and thus prevent extreme increases in latency because of undesired thrashing.

**Figure 5:**
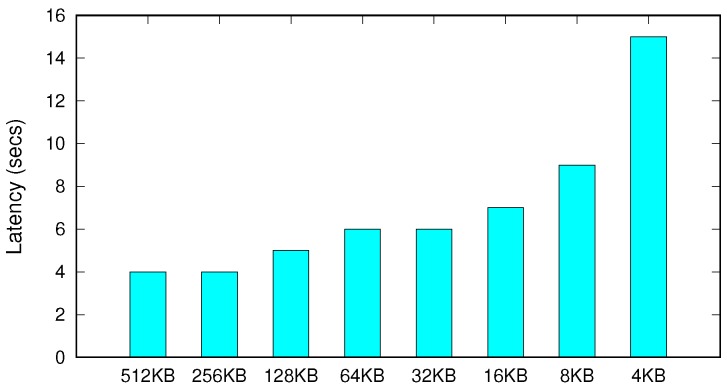
Latency of 4 GB storage write varying the write size.

The proposed cleaning scheme, termed proactive and coarse-grained cleaning, periodically examines the number of reclaimable dirty pages in the buffer. If the dirty pages have accumulated sufficiently to optimally perform storage write, SQLite forces them to storage in a batch. Because the performance of block storage devices has been optimized for throughput for last decades to alleviate their intrinsic long latency, a large-sized write can be performed orders of magnitude more efficiently than many of those that are small. To quickly verify the efficacy of our approach, we measured the latency of a 4 GB write to storage varying the I/O size from 4 KB to 512 KB. The performance was measured over the HFS+ file system mounted on 512 GB SSD (Solid-State Drive). Figure 5 shows that the I/O latency exponentially increases as the write granularity decreases. The latency of the fine-grained write (i.e., 4 KB) takes 3.5× higher than that of the coarse-grained write (i.e., 512 KB). These experimental results strongly support our proactive and coarse-grained cleaning scheme. Meanwhile, as shown in the figure, the performance variation decreases when the write size is larger than 32 KB, and the performance saturates at a size of 256 KB.

**Figure 6:**
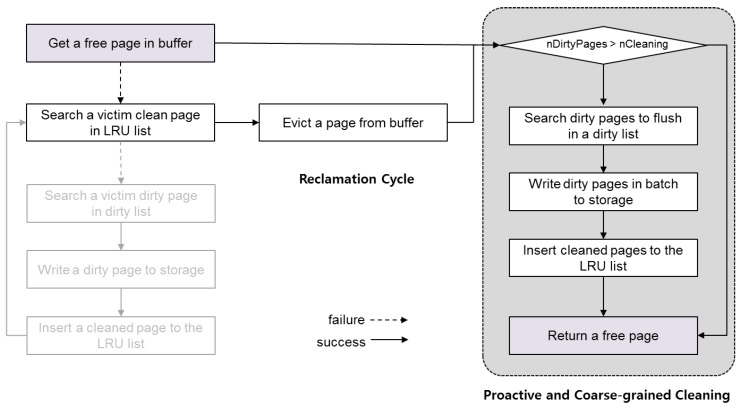
Memory reclamation scheme with proactive and coarse-grained cleaning.

Figure 6 shows the memory reclamation of SQLite when the proposed cleaning scheme is applied. In this architecture, whenever returning from the buffer page allocation routine, SQLite checks whether the number of reclaimable dirty pages is greater than the minimum size that can result in cleaning efficiency (denoted as nCleaning). If so, SQLite invokes the reclamation routine that flushes a group of dirty pages to storage in a batch and inserts them into the LRU list in the clean state. As a result, a group of reclaimable clean pages are available such that any subsequent requests for buffer space can be serviced without dirty page reclamation.

The benefits of proactive and coarse-grained cleaning seems straightforward, but implementing it efficiently and augmenting SQLite to make an effective use of it are not without challenges. To determine the best means of realizing our scheme, we explore two implementation methods: maintaining a dedicated thread for cleaning and performing cleaning in the context of a user thread.

The first implementation of our scheme is to rely on the background thread. By running a memory cleaning thread in the background, we can clean dirty pages in parallel with foreground request processing, hiding the cost of storage write from end users. In this version, SQLite creates a cleaning thread at the buffer deployment time and the thread sleeps until the dirty pages accumulate beyond the certain threshold. When the condition is satisfied, the thread wakes up and forces the dirty pages to storage altogether. Once the updates are successfully synchronized to storage, the associated dirty pages become a clean state and they are added into the LRU list. The cleaning thread stops running when the buffer is destroyed.

While the threaded version appears to be optimal in terms of user-perceived latency, this could be unrealistic in practice. The threaded version essentially needs a concurrency control mechanism to make concurrent updates to the shared data structures thread-safe. In SQLite, the buffer management system consists of multiple data structures that are tightly coupled to each other (e.g., B-tree, lists, and arrays). Therefore, implementing the non-blocking synchronization mechanism within the buffer management system is quite challenging. For this reason, the cleaning thread cannot help relying on the mutex lock, and the parallel execution of the cleaning thread can be highly limited because of locking contention, failing to interleave storage write with user requests.

Taking into consideration this challenge, we also study the non-threaded implementation of our proactive and coarse cleaning, which executes the cleaning routine in the context of user threads. This version has no potential to benefit from parallelism, but at least it can prevent the inter-thread communication overhead adding to the performance penalties.

One primary challenge of our scheme is how to set the threshold at which the cleaning operation is triggered. If the threshold is not sufficiently high, our cleaning scheme can adversely affect the performance because the cost of frequent invocation of the cleaning operation can outweigh the benefit of the coarse-grained cleaning scheme. To prevent this undesired circumstance, we empirically examined the performance of SQLite with respect to the threshold change, and the results are presented and discussed in Section 4.

## 4. Performance Evaluation

We evaluated the proposed memory reclamation scheme by means of a prototype implementation. We modified SQLite 3.29 to implement the two versions of the proactive and coarse-grained cleaning scheme. Our experiments were performed on an Intel Core i5 running at 3.3 GHz with 16 GB of DDR4 memory. The storage medium was backed by 512 GB SSDs. We measured the performance using an in-house benchmark that generates different sizes of transactions for performance evaluation. Our benchmark created a table with three columns, the primary key and two values, and inserted a series of data into the table by making use of SQLite APIs in C. The insert request consisted of an 8-length string primary key, an arbitrary string of 100 bytes as a value, and another integer type of value. The transaction referred to a set of insert requests that should be atomically processed. The transaction size can be configured by adjusting the number of requests. The in-memory buffer was set to use 100 pages (i.e., 400 KB), which is a default configuration in SQLite. For a comprehensive study, we considered the four SQLite journaling modes of SQLite described: DELETE, PERSIST, TRUNCATE, and WAL modes.

**Figure 7:**
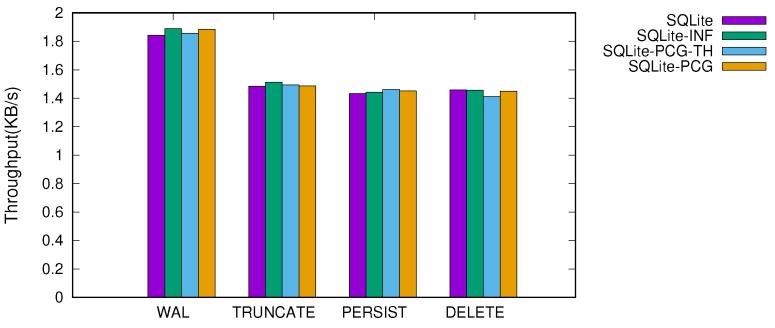
Throughput of 10 KB transaction in four different versions of SQLite.

**Figure 8:**
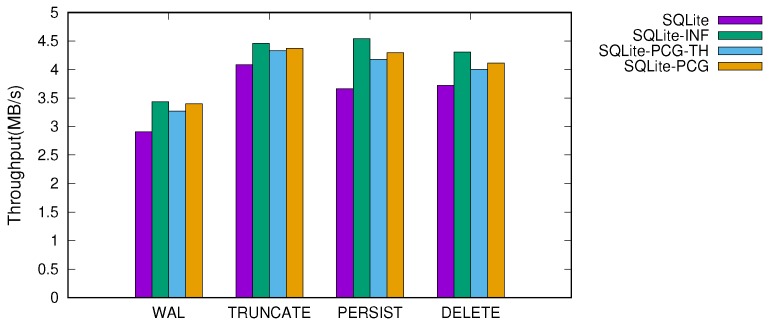
Throughput of 1 MB transaction in four different versions of SQLite.

Figure 7 and Figure 8 show the throughput of SQLite when performing 10 KB and 1 MB transactions, respectively. We studied four different version of SQLite; SQLite refers to as the original version of SQLite and SQLite-INF uses an infinite capacity of memory for the in-memory buffer. To this end, we configured SQLite-INF to use large memory as an internal buffer, which is greater than the total footprint of the transaction, so that no reclamation operation occurs during a transaction. SQLite-PCG-TH and SQLite-PCG represent when cleaning is performed with and without a background thread. For these two versions, we set the cleaning operation to be triggered when the number of reclaimable dirty pages exceeds 80% of the total buffer pages. This threshold was carefully configured through the empirical study, as discussed below. We executed tens of thousands of transactions, and report the average throughput.

When the transaction size is smaller than the in-memory buffer size (e.g., a 10 KB transaction), the performance variation is small across different versions of SQLite. Under these workloads, the impact of the reclamation scheme is less than that of the journaling mechanisms in SQLite. However, when the large transactions that exceed the in-memory buffer size are issued, the dynamic memory reclamation is triggered and thus the efficiency of the memory reclamation scheme significantly affects the performance. The original SQLite that pursues fine-grained flushing of the dirty pages shows inferior performance, less than that when using an infinite memory by up to 19.4%.

When the proactive and coarse-grained cleaning scheme is applied (i.e., SQLite-PCG and SQLite-PCG-TH), however, the performance improves by 6–17% across all configurations. The merged write enhances significantly I/O efficiency and thus provides the performance near that of SQLite-INF. Compared to SQLite-INF, the performance degradation of SQLite-PCG-TH and SQLite-PCG are within 9% and 5.4%, respectively.

One counter-intuitive result is that the performance variation is marginal when using a thread or not. In terms of concurrency, the blocking concurrency control of the in-memory buffer highly constrains the concurrent execution of the background thread, failing to achieve an effective benefit from parallelism. Meanwhile, the thread maintenance overhead is mostly amortized by the batches of writes because the cleaning intervals are not that short in practice.

**Figure 9:**
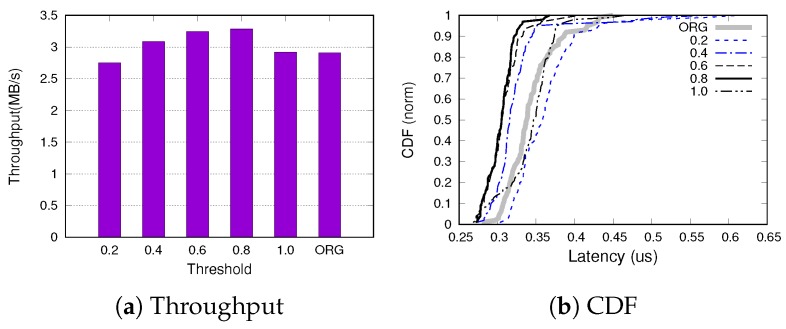
SQLite Performance varying the threshold value.

We investigated the performance of SQLite with respect to the different threshold values at which the cleaning operation is invoked in our proposed scheme. We represent the threshold as the ratio of dirty pages in the buffer and measure the performance varying the threshold value from 0.2 to 1. Figure 9a shows the SQLite throughput as a function of the threshold value and Figure 9b shows the CDF (Cumulative Distributed Function) of transaction latency for the same threshold spectrum. This result is obtained using SQLite-PCG running in the WAL journaling mode.

When the threshold is set to the small value (i.e., 0.2), the proactive coarse-grained cleaning scheme adversely affects the performance, delivering lower throughput and higher latency than original SQLite. In this setting, the benefit of coalescing writes is not sufficient to offset the additional overhead of our scheme. However, as the threshold increases, our scheme outperforms the original SQLite both in terms of throughput and latency. The performance benefit saturates when the threshold is 0.6, which is consistent with the results shown in Figure 5. One interesting observation is that when the threshold is set to 1, the performance is nearly identical to that of the original version. According to our analysis, in this configuration, the original reclamation scheme is executed before our cleaning and cleans one dirty page. This behavior never results in the ratio of dirty pages becoming 1 and thus our cleaning scheme is not invoked. Based on these experimental results, we set the threshold to 0.8 in this study as it shows the best performance across all values.

## 5. Related Work

### 5.1. SQLite as Healthcare Data Storage

With the activation of data-driven healthcare services, the techniques for efficient exchange and storage of healthcare data have been actively explored in many studies. Doukas et al. proposed pervasive healthcare data storage that runs over the cloud systems and is accessible from the mobile devices [6]. They stored the health data in an SQLite file format in cloud storage and then used a mobile device when needed so as to readily query the data. This pervasive data storage system can be utilized in telemedicine, pateient monitoring, and emergency response and monitoring. Maglogiannis et al. noted that large volume of medical data such as magnetic resonance imaging (MRI) has a long transmission time and proposed a data compression technique to reduce the number of transmitted data [18]. A robot-based biofeedback system for elderly people was presented by Lopez-Samaniego et al. that relies on SQLite for healthcare data management [8]. Hu et al. developed a prescribed fire information database such that users can easily access the environmental information that affects their health such as air quality and an analysis of exposure to smoke, managing their activities related to the health [19]. Chen et al. proposed a fast healthcare data service system that stores medical information into a gateway, thereby providing a quick response and enabling fast decision making, without reaching a remote server in a cloud medical information system [5]. In this system, the gateway also makes use of SQLite to provide a medical information database. Because the proposed memory reclamation policy in this paper eliminates the unpredictable delay of SQLite using a proactive approach, it will serve well these medical systems that need real-time decision-making and fast responsiveness. SQLite is not only used as a backend database component in the blockchain based health data management systems [20], but also used in real-time patient monitoring to store data in a local database [21]. In addition, SQLite serves as a database engine within an audit system that detects data corruption such as healthcare data breaches in web-based data service platforms [22].

### 5.2. SQLite Performance Optimization

Many studies have been performed to resolve the performance problems caused by journaling in SQLite. Park et al. noted that coarse-grained logging in the current SQLite incurs serious write amplification, such as redundant writes of several physical pages even for a single small transaction [23]. To address this problem, they presented a statement-level logging approach that eliminates the superfluous writes of coarse-grained logging and effectively enhances SQLite write performance. Kang et al. also observed that the WAL of SQLite severely sacrifices performance for reliability, dramatically increasing the write traffic [24]. They overcame this challenge by using a write-efficient journaling technique based on data compression. Oh et al. observed that SQLite transactions are mostly small sized on mobile devices; the automatic commit operation for these transactions subsequently forces numerous small and random writes into storage [25]. These workloads are exceedingly harmful for SSDs, as they are likely to be a primary cause of serious write amplification in the GC (Garbage Collection). They argued for the adoption of non-volatile memory for transaction logging because it is not only able to quickly handle small-sized writes but is also persistent. Luo et al. [26] and Lee et al. [27] also revealed that the double-write penalty of write-ahead logging is very costly in actual systems. They attempted to implement a non-volatile page cache that persistently holds updates within the main memory, thus obviating the need for logging.

There are some studies that have improved SQLite performance through the storage interface refactoring. Min et al. optimized the FTL(Flash Translation Layer) controller of an SSD such that it supports atomic updating of a series of data within the SSD [28]. By exporting this feature as a storage interface, they enabled SQLite to provide transactional writes without write-twice logging. A study by Yeon et al. also presented a new storage interface that individually synchronizes transactions generated by database systems such as SQLite so as to isolate the performance penalty of each transaction from the others [29].

### 5.3. Database Buffer Management

Previous research of SQLite has mainly focused on enhancing storage-side components such as I/O stack optimization and write-efficient logging, neglecting the effects of the in-memory ingredients of SQLite. Indeed, the flushing of dirty buffers via a background thread is a common technique in state-of-the-art DBMS such as MySQL and Oracle [30], and various buffer replacement techniques have been studied in the database community for decades [12]. The background thread approach suits well a server-side DBMS because it has abundant resources and a large size of buffer (i.e., GBs), it is possible to execute foreground request processing and background threads in parallel without excessive performance penalty. For example, the Innodb database engine for MySQL maintains multiple buffer instances when the memory size is greater than 1 GB, which can be simultaneously accessed, enabling interleaving of different jobs [31].

In contrast to these server-side databases engines, as SQLite typically uses a small memory buffer size, and pursues the code simplicity in its design, the SQLite buffer management scheme has not been receiving much attention until now. Despite numerous previous studies on SQLite, they have mostly focused on the optimization techniques for improving storage I/O performance, and while a few recent works discussed the SQLite buffer management scheme, they studied the buffer structures to exploit emerging memory devices such as phase change memory and SSDs [25]. As opposed to these studies, our work identifies a drawback of the current SQLite in-memory buffer management mechanism and overcomes it by presenting a judicious memory reclamation scheme. Given that memory capacities are increasingly growing and the in-memory components play a great role in modern computing systems, our work is quite timely and worthwhile in practice. To the best of our knowledge, this is the first study that explores the buffer reclamation schemes to prevent unexpected delays of large transactions in SQLite.

## 6. Conclusions

This study discovered the abnormal delay during processing of large-sized transactions in SQLite, which is extensively used in various data storage systems including healthcare and medical data management. We observed that the current fine-grained buffer reclamation scheme of SQLite provides poor performance for large-sized transactions by significantly decreasing the I/O efficiency with individual flushing of the dirty pages. This drawback causes a long delay during transaction processing of SQLite, making fast decision making and the real-time medical data services in an emergency a challenge. To remedy this problem, we present a proactive and coarse-grained cleaning scheme, which merges several small writes as a single write stream, thereby minimizing the overhead of storage write for reclamation. The proposed scheme was implemented in SQLite 3.29, and the performance evaluation using a prototype implementation demonstrated that our scheme improves I/O performance by 13% on average, compared to the original version of SQLite. By eliminating an unexpected delay in SQLite, the proposed memory reclamation scheme is able to invariably offer a fast response, which is critical in real-time healthcare database systems.
